# A Multiuser Detector Based on Artificial Bee Colony Algorithm for DS-UWB Systems

**DOI:** 10.1155/2013/547656

**Published:** 2013-07-31

**Authors:** Zhendong Yin, Xiaohui Liu, Zhilu Wu

**Affiliations:** School of Electronics and Information Engineering, Harbin Institute of Technology, Harbin 150001, China

## Abstract

Artificial Bee Colony (ABC) algorithm is an optimization algorithm based on the intelligent behavior of honey bee swarm. The ABC algorithm was developed to solve optimizing numerical problems and revealed premising results in processing time and solution quality. In ABC, a colony of artificial bees search for rich artificial food sources; the optimizing numerical problems are converted to the problem of finding the best parameter which minimizes an objective function. Then, the artificial bees randomly discover a population of initial solutions and then iteratively improve them by employing the behavior: moving towards better solutions by means of a neighbor search mechanism while abandoning poor solutions. In this paper, an efficient multiuser detector based on a suboptimal code mapping multiuser detector and artificial bee colony algorithm (SCM-ABC-MUD) is proposed and implemented in direct-sequence ultra-wideband (DS-UWB) systems under the additive white Gaussian noise (AWGN) channel. The simulation results demonstrate that the BER and the near-far effect resistance performances of this proposed algorithm are quite close to those of the optimum multiuser detector (OMD) while its computational complexity is much lower than that of OMD. Furthermore, the BER performance of SCM-ABC-MUD is not sensitive to the number of active users and can obtain a large system capacity.

## 1. Introduction

Since the concept of ultra-wideband (UWB) technology was put forward by FCC in the 1990s, UWB technique has drawn a lot of attention in the theoretic research, industrial application, and many other areas because of its attractive features such as high data transmission rate, low power density, high interference resistance, and strong multipath resolution [1–3].

Multiuser detection (MUD) is a method to eliminate the effect of multiple access interference (MAI). The multiple accesses are commonly divided into two paths: time hopping (TH-UWB) and direct sequence (DS-UWB) [4]. In recent years, the increase in demand for multiple access applications with high data transmission rates has prompted the development of multiuser detection (MUD) techniques in such systems to suppress the MAI and improve the system performance. Optimum multiuser detection (OMD) was provided by Verdu in 1986 [5]; OMD makes the BER performance of multiple access system approximate to single-user system. But it has a high computational complexity, so it has poor real-time characteristic confines in engineering. Therefore, suboptimal detectors which may approximate OMD's BER performance with an acceptable computational complexity have become a focus of research. A code-aided interference suppression method was introduced for NBI restriction in DS-UWB systems [6]. A multiuser frequency-domain (FD) turbo detector was employed which combines FD turbo equalization schemes with soft interference cancellation [7]. Adaptive MUD methods using the recursive least square (RLS) principles were proposed in [8, 9]. However, the tradeoff problem between BER performance and computational complexity still exists.

Swarm intelligence is a research branch that models the population of interacting agents or swarms that are able to self-organize. Several modern heuristic algorithms have been developed for solving combinatorial and numeric optimization problems [10]. Artificial Bee Colony (ABC) algorithm is an optimization algorithm based on the intelligent behavior of honey bee swarm [11, 12], and some extended and efficient ABC algorithms are presented to improve the performance of ABC [13, 14].

It has been demonstrated that ABC algorithm for numerical optimization problems has more superior performance than those based on heuristics algorithm [15]. ABC algorithm has been widely used in neural network training practice [16], path optimization [17], and back analysis [18]. However, no literatures have been reported that ABC was used in the multiuser detection (MUD) fields. In order to possess a good BER performance and a low time complexity, we investigate an efficient multiuser detector by selection of initial states based on code mapping for the Artificial Bee Colony algorithm (SCM-ABC-MUD) in DS-UWB systems. As a kind of swarm intelligence methods, ABC is selected here for its significant ability to search for the global optimal value and to adapt its searching space automatically [19, 20]. And its basic motivation is to find the global optimum by simulating the bees' behaviors [21]. This proposed algorithm makes use of the thought of ABC's iterative optimization and the result of the suboptimal detectors based on code mapping. Simulation results show that the BER performance and near-far effect (NFE) resistance capability of this algorithm are better than those of matched filter (MF), DEC, and MMSE detectors and even close to OMD.

The paper is organized as follows. In Section 2, the MUD model is introduced and a suboptimal detector based on code mapping is proposed. Then in Section 3, the basic principles of the Artificial Bee Colony (ABC) algorithm and the proposed SCM-ABC-MUD algorithm are illustrated. In Section 4, simulation experiments that compare the performance of different MUD algorithms are analyzed, followed by conclusions given in Section 5.

## 2. MUD Models and Methods

### 2.1. Multiuser DS-UWB System Model

 In theory, a *K*-user synchronous DS-UWB system under the additive white Gaussian noise (AWGN) channel is considered which is not subjected to the frequency selective multipath. And assume each user employs the binary phase-shift key (BPSK) modulation. Then the *k*th user's transmit signal can be written as

(1) xk(t) =∑i=1M∑j=0Nc−1dk(i)ck(t−(i−1)Ts)p(t−(i−1)Ts−jTc),

where *M* is the length of bits per packet and BPSK symbols  *d*_*k*_(*i*)∈{−1,1}_*i*=1_^*M*^ are spread with the specific PN codes *c*_*k*_(*t*), which are the binary bit stream valued only by −1 or 1. *T*_*s*_ is the symbol duration, *T*_*c*_  is the pulse repetition period, *N*_*c*_ equals to *T*_*s*_/*T*_*c*_, and *p*(*t*) represents the transmitted pulse waveform generally characterized as the second derivative of Gaussian pulse

(2) $$\begin{array}{c} p\left(t\right)=\left[1-4\pi {\left(\frac{t-t_{d}}{\tau_{m}}\right)}^{2}\right]\exp\left[-2\pi {\left(\frac{t-t_{d}}{\tau_{m}}\right)}^{2}\right], \end{array}$$

where *t*_*d*_  and *τ*_*m*_  are the pulse center and the pulse shape parameter.

The total received signal composed by different signals of all users is

(3) $$\begin{array}{c} r\left(t\right)=v\left(t\right)+n\left(t\right)=\sum_{k=1}^{K}A_{k}x_{k}\left(t\right)+n\left(t\right), \end{array}$$

where *A*_*k*_ is the amplitude of the *k*th received signal and *n*(*t*) is zero-mean additive white Gaussian noise with the unilateral power spectral density of *N*_0_.

### 2.2. Classical Multiuser Detectors

#### 2.2.1. Matched Filters (MFs) 

The traditional receiver of a DS-UWB system consists of a pulse demodulator and a set of matched filters (MFs) corresponding to each user. Let the output of a bank of single-user MFs be a *K*-dimensional vector  **y** = [*y*_1_, *y*_2_,…,*y*_*K*_]^*T*^, the vector **b** = [*b*_1_, *b*_2_,…,*b*_*K*_]^*T*^ represent the output of sign detectors, the vector **d** = [*d*_1_, *d*_2_,…,*d*_*K*_]^*T*^ denotes the correct bits of each user, and the vector **n** = [*n*_1_, *n*_2_,…,*n*_*K*_]^*T*^ denotes the output of noise from matched filters which is a zero mean Gaussian random. So, the output of the MFs can be represented as follows:

(4) $$\begin{array}{c} y\;=\;RAb\;+\;n, \end{array}$$

(5) $$\begin{array}{c} b=\mathrm{sgn}\left(y\right), \end{array}$$

where *R* = (*r*_*ij*_)_*K*×*K*_ denotes the cross-correlation matrix, in which *r*_*ij*_ = ∑_*l*=0_^*N*_*c*_−1^ *c*_*i*_(*l*)*c*_*j*_(*l*), and **A** = diag⁡(*A*_1_, *A*_2_,…, *A*_*K*_) in which the diagonal element *A*_*k*_  (*k* ∈ [1, *K*], *k* ∈ *N*) represents the signal amplitude of the *k*th user.

#### 2.2.2. Optimum Multiuser Detection (OMD)

 According to the theory of OMD, the optimum detection result satisfies the following expression:

(6) $$\begin{array}{c} b_{\text{OMD}}=\arg\left\{\underset{b\in \left\{-1,1\right\}}{\max}\left(2b^{T}Ay-b^{T}ARAb\right)\right\}. \end{array}$$

It is known that the selection of this optimal solution **b**_OMD_ in the *K*-dimensional Euclidean solution space is generally a nondeterministic polynomial hard problem, but the computational complexity of the OMD method is O(*K* ^2^), and *K* is the number of active users.

#### 2.2.3. Suboptimal Multiuser Detection Based on Code Mapping (SCM) 

In order to get a suboptimal solution, the candidate bits set output from the matched filters mapped to a one-dimensional feature space using a mapping function.

Let

(7) $$\begin{array}{c} F\left(b\right)=\frac{1}{2}b^{T}ARAb-b^{T}Ay. \end{array}$$

According to (6), if the elements in **b** are all right, the value of *F*(**b**) will achieve the minimum. Making a partial derivation of (7), we get

(8) $$\begin{array}{c} \frac{\partial F}{\partial b}=Hb-Ay. \end{array}$$

By expanding (8), we get a *K*th-order linear equations given as follows:

(9) $$\begin{array}{c} \frac{\partial F}{\partial b_{1}}=\sum_{j=1}^{K}A_{1}A_{j}r_{1j}b_{j}-A_{1}y_{1},\\ \frac{\partial F}{\partial b_{2}}=\sum_{j=1}^{K}A_{2}A_{j}r_{2j}b_{j}-A_{2}y_{2},\\ \vdots \\ \frac{\partial F}{\partial b_{K}}=\sum_{j=1}^{K}A_{K}A_{j}r_{Kj}b_{j}-A_{K}y_{K.} \end{array}$$

Now, we take into account that MAI is the main interference resource which largely affects the performance of the system. Let *L*(*b*_*i*_) = ∑_*j*=1_^*K*^ *A*_*i*_ *A*_*j*_ *r*_*ij*_ *b*_*j*_ − *A*_*i*_ *y*_*i*_, *i* = 1,2,…, *K*. Bringing the candidate codes set **b**  into (9), there are two situations illustrated as follows.

(1) No wrong code in **b**. Based on the theory of extreme value, if MAI is the only interference resource without AWGN and the elements in **b** are all correct, the result of (9) strictly equals 0. And, in the condition of high SNR, bringing (4) to *L*(*b*_*k*_), we can see that *L*(*b*_*k*_) = −*A*_*i*_ *n*_*i*_ ~ *N*(0, *A*_*i*_^2^ *N*_*c*_ *N*_0_/2)(*k* = 1,2,…, *K*) when there is no error bit in the candidate codes set **b**.
(2) Wrong codes exist in **b**. Supposing *b*_*i*_  (*i* ∈ [1, *K*], *i* ∈ *N*) is the wrong code (in other words, −*b*_*i*_ is the correct code), the other codes are correct. Substituting it to the *i*th equation of (9), we get
  (10) L(bi)=∑j=1j≠iKAiAjrijbj−Aiyi+Ai2riibi=∑j=1j≠iKAiAjrijbj−Aiyi+Ai2rii(−bi)+2Ai2riibi=2Ai2Ncbi−Aini.
 As for *k* which does not equal *i*, we get
  (11) $$\begin{array}{c} L\left(b_{k}\right)=2A_{i}A_{k}r_{ik}b_{i}-A_{k}n_{k},\;\;k=1,2,\ldots ,K,\;\;k\neq i. \end{array}$$

According to (10) and (11), it can be seen when the *i*th user's code is wrong, |*L*(*b*_*i*_)|≫ | *L*(*b*_*k*_)|, *k* = 1,2,…, *K*, *k* ≠ *i*. Therefore, the function *L*(**b**) can obviously differentiate the wrong codes and the right codes through the absolute value of it. In addition, *L*(**b**) is a *K*th-order linear equation which can get the result of *L*(**b**) without complex computations.

**Figure 1:**
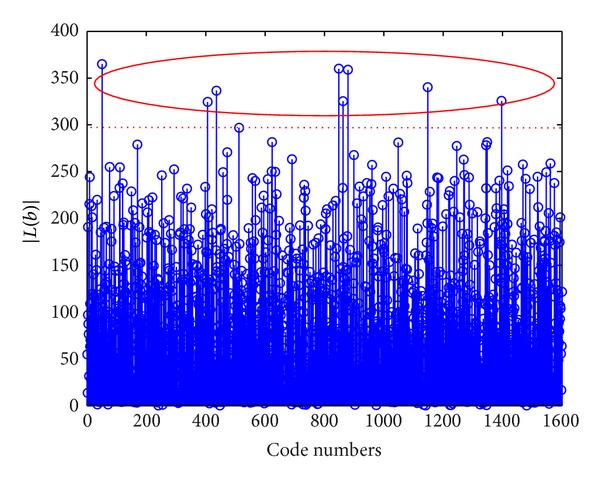
Relationship between code numbers and mapping function |*L*(**b**)|.

It is accessible to map *b* into a one-dimensional feature space |*L*(**b**)| to identify the wrong codes in the candidate set.Figure 1 shows an example of the feature space mapping in the scenario of 10 users, with 6 dB signal-to-noise ratio (SNR).

In Figure 1, it is clear that the wrong codes and the right ones have a significant difference in the feature space mapped by |*L*(**b**)|. Almost all the values of |*L*(**b**)| corresponding to the wrong codes are greater than 280; in contrast the values of the right ones are smaller than 280. *C*-means clustering approach [22] is used to classify the candidate codes into right bits and wrong ones. The *C*-means clustering is a popular unsupervised clustering algorithm based on the partition of data, which can finish the classification without training samples.

In conclusion, the whole proposed approach can perform the multiuser detection with 3 steps. First, map the candidate bits set **b**  into a one-dimensional feature space by mapping function |*L*(**b**)|. Second, aggregate and pick out the wrong bits among the candidate bits set by code clustering. And finally, correct the wrong bits to obtain a suboptimal solution.

## 3. The Proposed SCM-ABC-MUD Algorithm

### 3.1. The Basic Principle of ABC Algorithm

In the ABC algorithm, the colony of artificial bees consists of three kinds of bees: employed bees, onlookers, and scouts. In the algorithm, the number of employed bees is equal to the number of food sources around the hive. The employed bee whose food source is exhausted by the employed and onlooker bees becomes a scout. The position of a food source represents a possible solution of the optimization problem, and the nectar amount of a food source corresponds to the quality (fitness) of the associated solution. The number of the onlooker bees or the employed bees is equal to the number of food sources in the population.

The general scheme of the ABC algorithm is as follows.

**Step 1**

The first step is initialization. In this step, a set of food source positions is generated and distributed randomly. After initializing the solution population, the fitness value of each food source position is evaluated. In ABC algorithm, the fitness of each solution is the value of objective function. The ABC has some control parameters: food sources number (the number of food sources equal to the employed bees); limit, the value of predetermined number of cycles; and MCN, the maximum cycle number. Initially, the values of these control parameters are assigned.

**Step 2**

After initialization, the population is evaluated and is subjected to repeated cycles of the search processes of the employed bees, the onlooker bees, and scout bees. Employed bees search for new food sources  (*V*_*m*_) having more nectar within the neighborhood of the food source (*X*_*m*_) in their memory. They find a neighbor food source and then evaluate its profitability (fitness). They can determine a neighbor food source *V*_*m*_ using the formula given by

(12) $$\begin{array}{c} v_{mi}=x_{mi}+\theta_{mi}\left(x_{mi}-x_{ki}\right), \end{array}$$

where *x*_*k*_ is a randomly selected food source, *i* is a randomly chosen parameter index, and *θ*_*mi*_ is random number between [−1,1].

**Step 3**

After producing the new food source *V*_*m*_, its fitness is calculated and a greedy selection is applied between *V*_*m*_ and *X*_*m*_. If the fitness of the new food source position is better than the old food source position, the employed bee moves from the old food source position to the new food source position, and the new food source takes place of old one in the population and becomes a new member.

The fitness value of the solution, fit_*m*_(*X*_*m*_), might be calculated for minimization problems using the following formula:

(13) $$\begin{array}{c} \text{fi}{\text{t}}_{m}\left(X_{m}\right)=\{\begin{array}{cc} \frac{1}{1+f_{m}\left(X_{m}\right)}, & \text{if}\;f_{m}\left(X_{m}\right)\geq 0,\\ 1+\text{abs}\left(f_{m}\left(X_{m}\right)\right), & \text{if}\;f_{m}\left(X_{m}\right)\leq 0, \end{array} \end{array}$$

where *f*_*m*_(*X*_*m*_) is the objective function value of solution *X*_*m*_.

**Step 4**

Unemployed bees consist of two groups of bees: onlooker bees and scouts. Employed bees share their food source information with onlooker bees waiting in the hive, and then onlooker bees probabilistically choose their food sources depending on the probability values calculated using the fitness values provided by employed bees.

Calculate the probability values *P*_*i*_ for the solutions using fitness of the solutions by

(14) $$\begin{array}{c} P_{i}=\frac{\text{fi}{\text{t}}_{m}\left(X_{m}\right)}{\sum_{m=1}^{\text{SN}}\text{fi}{\text{t}}_{m}\left(X_{m}\right)}, \end{array}$$

where SN represents the number of the food sources position.

After a food source *X*_*m*_ for an onlooker bee is probabilistically chosen, a neighborhood source *V*_*m*_ is determined by Step 2, and its fitness value is computed. As in Step 3, a greedy selection is applied between *V*_*m*_ and *X*_*m*_. Hence, more onlookers are recruited to richer sources, and positive feedback behavior appears.

**Step 5**

If a predetermined number of trials cannot further improve a candidate solution represented by a food source then that food source, is considered abandoned and the employed bee associated with that food source becomes a scout. The new solution discovered by the scout can be defined by

(15) $$\begin{array}{c} x_{mi}=l_{i}+\text{rand}\left(0,1\right)*\left(u_{i}-l_{i}\right), \end{array}$$

where  *l*_*i*_ and *u*_*i*_ are the lower and upper bounds of the parameters *x*_*mi*_. The abandoned food source is replaced by the randomly new food source.

**Step 6**

If a termination condition is reached, the process is stopped and memorizes the best solution achieved so far. Otherwise the algorithm returns to Step 2.

The general algorithmic structure of ABC optimization approach can be summarized as follows [23]:

- *Initialization Phase*
- *Repeat*
   Employed Bees Phase
   Onlooker Bees Phase
   Scout Bees Phase
   Memorize the best solution achieved so far
- *Until* (Cycle = Maximum Cycle Number).

### 3.2. The Discretization of Behavior Models

The mathematical model of OMD is considered as a combinatorial optimization problem, and ABC has a strong global searching capability to solve this problem. The optimization function for OMD is shown as (6), which is a discrete optimization function; for this reason, the behavior models of ABC should be discretized.

The discretization is defined as follows.

1. The expression of artificial bee's state. In this solution space, the state of each artificial bee is encoded by −1 or +1. If there are *K* active users in this DS-UWB MA system, thus the state is a *K*-dimensional vector, like  *X*_0_ = (*x*_1_, *x*_2_,…,*x*_*K*_)^*T*^, where *x*_*i*_ ∈ {−1, + 1} and *i* = 1,2,…, *K*.
2. The food quality or the fitness function for artificial bees is the criterion of OMD in (6).
3. *X*_*c*_  is the new food sources for each step; if the element of *X*_*c*_  is  *x*_*ci*_ > 0, then assign *x*_*ci*_ = +1; otherwise, *x*_*ci*_ = −1.
4. Initialization. The initial state of each artificial bee is selected randomly in the discrete space with 2^*K*^ likely solutions. But the ABC is an iterative optimization algorithm, which means that the selection of initial states has a great effect on the iteration times and the convergence rate. In this paper, we choose a suboptimal multiuser detection based on code mapping (SCM) and its variations as initial state values of artificial bees, which can ensure the artificial bees gather near the optimal value of the optimization problem because the suboptimal solution is quite close to the optimal value in the solution space. Besides, this selection method of initial states can speed up the convergence rate and reduce the iteration times for optimization.

### 3.3. The Specific Steps of the Initial Value Selection

The specific steps of the initial value selection are given as follows.

**Step 1**

Execute matched filter detection and code mapping to get a suboptimal solution first. Assign the result  *b*_1×*K*_ = (*b*_1_, *b*_2_,…, *b*_*K*_) to the first artificial bee as its initial state value, where *b*_*i*_ ∈ {−1, + 1} and *i* = 1, 2, …, *K*.

**Step 2**

Change an element *b*_*i*_ of the result  *b*_1×*K*_ randomly, *b*_i_* = −*b*_*i*_, and then assign this new result *b*_1×K_* which changes from *b*_1×*K*_ to the second artificial bee as its initial state value.

**Step 3**

Repeat Step 2 and assign these new results to other artificial bees.

**Step 4**

**Figure 2:**
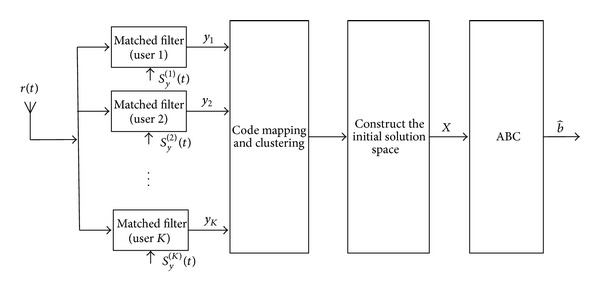
General schematic diagram of the SCM-ABC-MUD detector.

If all artificial bees have been assigned initial state values, the procedure of initial value selection comes to the end. And we can start the ABC to research the optimal value for MUD. In consideration of the previous statements, the overall structure of this proposed SCM-ABC-MUD detector is shown in Figure 2.

Figure 2 shows the block diagram of the algorithm. The implementation of this detector can be summarized as follows: firstly, the output of a bank of matched filter receivers is fed to suboptimal detectors based code mapping and clustering; secondly, the detection results of these suboptimal detectors are used to construct an initial solution space; finally, the ABC is executed in this space.

## 4. Simulation and Discussion

In order to analyze the proposed SCM-ABC-MUD algorithm, Monte Carlo simulations are utilized and the values of these majority parameters are assigned as Table 1. 

### 4.1. The BER Performance versus SNR

**Figure 3:**
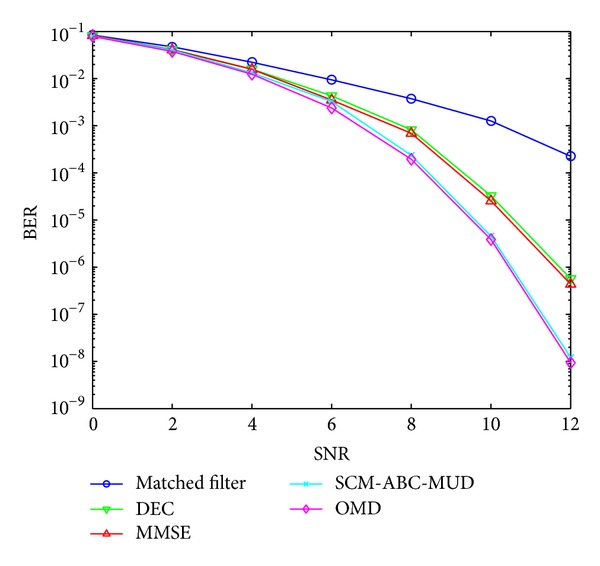
BER performance versus SNR.

The BER versus SNR curves with perfect power control in the AWNG are depicted in Figure 3. And there are 10 users in the system. As for ABC, we fix the number of initializing food sources (equal to employed bees) that is 3; limit, the value of predetermined number of cycles, that is 3; and MCN, the maximum cycle number, that is 20.

The performances of matched filter (MF), decor relating (DEC) [24], minimum mean square error (MMSE) [25], SCM-ABC-MUD, and OMD detectors are compared and depicted in Figure 3.

Figure 3 shows that the BER performance of SCM-ABC-MUD is superior to those of other suboptimal detectors including MF, DEC, and MMSE, and it even coincides with that of OMD. The reason is that this proposed SCM-ABC-MUD algorithm can make a search within a simplified solution space constructed by the solutions of these suboptimal detectors rather than a random search.

### 4.2. The BER Performance versus User Numbers *K*

**Figure 4:**
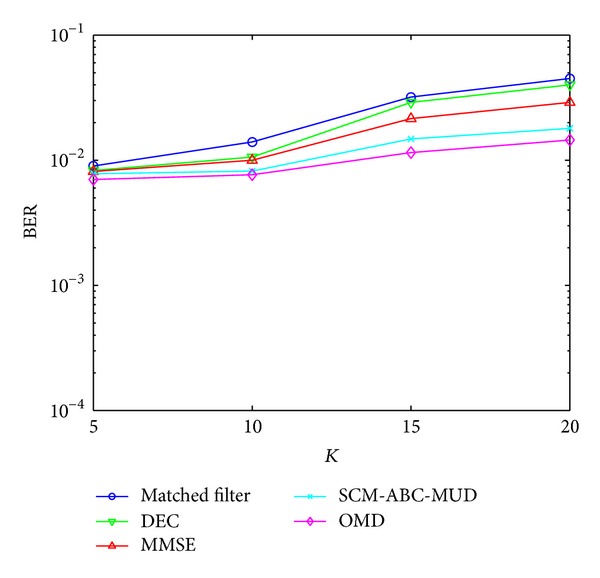
BER performance versus user numbers *K*.

The BER performances of these detectors with different number of active users *K* are analyzed in this experiment. The SNR of the AWGN channel is 5 dB. And the control parameters of ABC are same as the BER performance versus SNR.  Figure 4 shows the result of this experiment. According to Figure 4, the performance of SCM-ABC-MUD is the best of the suboptimum MUD except OMD. While the number of users increases, ABC needs a new group of control parameters. However, in this experiment, they are unchanged, resulting in the performance difference between SCM-ABC-MUD and OMD.

### 4.3. The NFE Resistant Capability Comparison

**Figure 5:**
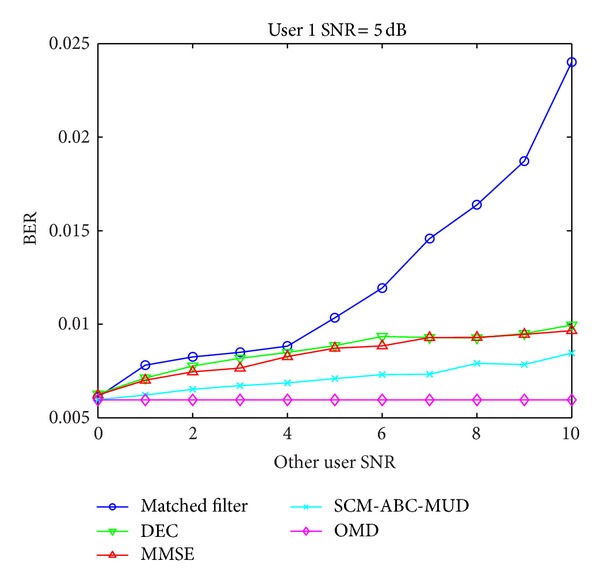
Near-far effect resistant of different MUD algorithms.

 In this experiment, the SNR of the first user's received signal is assigned at 5 dB all the time, while the SNR of other users changs from 0 dB to 10 dB with per step of 1 dB. Besides, other parameters are set as same as those in the BER performance versus SNR.  Figure 5 shows the first user's BER performances of different MUD detectors. It can be seen from Figure 5 that OMD has the best near-far effect resistant ability, and the SCM-ABC-MUD is very close to OMD.

### 4.4. BER Performance versus Different Initial States

**Figure 6:**
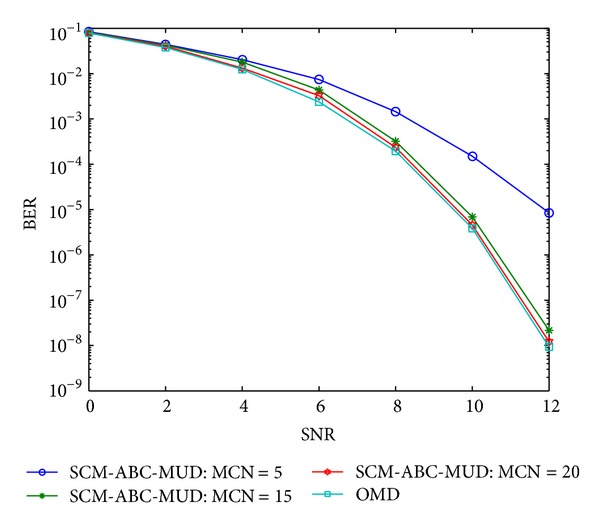
The BER performance with different iteration times of SCM-ABC-MUD.

**Figure 7:**
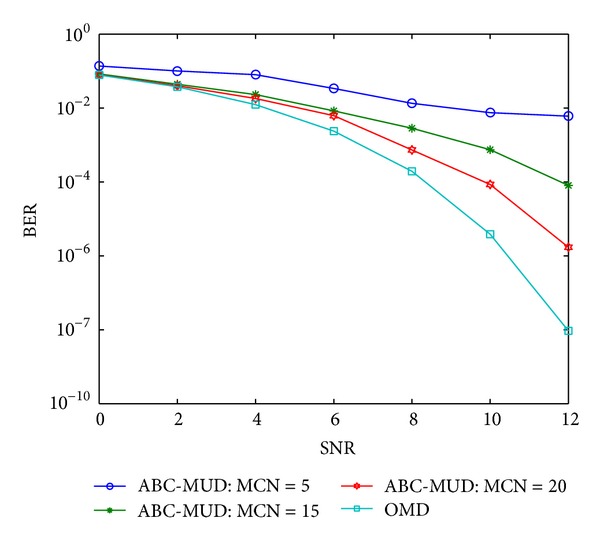
The BER performance with different iteration times of ABC-MUD.

 As an iterative optimization algorithm, the convergence rate is as important as the optimization capability. And the selection of initial states has a great effect on the iteration times and the convergence rate. In this experiment two initial state values of artificial bees are employed as a comparison. One has random initial state values which is named as ABC-MUD; the other is SCM-ABC-MUD which is assigned a group of initial state values using the suboptimal solution of the code mapping-based multiuser detection. The BER performance is studied for both algorithms with the maximum cycle number (MCN) of 5, 15, and 20, respectively. Figures 6 and 7 are the convergence rates of ABC-MUD and SCM-ABC-MUD separately.

From Figures 6 and 7, the BER performance of SCM-ABC-MUD is much better than ABC-MUD with the same iteration times, and the former can achieve OMD's BER performance with 20 times iteration which is the global optimal solution. It can be concluded that the convergence rate of SCM-ABC-MUD is much quicker than that of ABC-MUD. Meanwhile, ABC-MUD only improves its BER performance a little than matched filter (shown in Figure 3). It is because that SCM-ABC-MUD makes full use of the suboptimal solution, the initial value of SCM-ABC-MUD is more close to OMD than ABC-MUD, and so it can achieve the optimal value of OMD much quicker than ABC-MUD.

### 4.5. The Computational Complexity of the Proposed SCM-ABC-MUD

OMD can obtain the best BER performance while the computational complexity performance is too expensive to be implemented in practice. However, the computational complexity of SCM-ABC- MUD is much less than that of the OMD. In a *K*-user DS-UWB system, to detect the *K*-user vector **b**, the computational complexity of iterations of the OMD is 2^*K*^. The computational complexity of the proposed method includes three parts. The first part is the computational complexity of the code mapping; according to Section 2.2.3, the mapping method is just a calculation of *L*(**b**), so the computational complexity of the first part can be neglected. The second part is the computational complexity of *C*-means clustering; the complexity of iterations of the code clustering is *K*. The third part is the complexity of the ABC algorithm. It depends on the convergence rate of SCM-ABC-MUD. In Section 4.4, we have obtained a conclusion that the SCM-ABC-MUD can achieve convergence with a much fewer iterations than OMD. Let the normalized operation time of per vector **b** using matched filter detector equal to 1 in the condition of 10 users. The simulation parameters are the same as Section 4.1.  Table 2 lists the relative operation time using OMD, SCM-ABC-MUD, and matched filter.

From Table 2 we can see that the computational complexity of the SCM-ABC-MUD is in the same order of magnitude to the MMSE and DEC and far lower than OMD. This is because the iteration of SCM-ABC-MUD will be converged very soon and costs little quantity of computation. Hence, we can get a conclusion that the SCM-ABC-MUD can get good BER performance with low computational complexity.

## 5. Conclusions

In this paper, we firstly employed the Artificial Bee Colony algorithm in the DS-UWB MUD. In consideration of the high computational complexity of OMD, the proposed MUD is a hybrid method which combines ABC algorithm and a suboptimal solution of the code mapping-based MUD. First, the bits set output from the matched filters is mapped into a one-dimensional feature space to obtain a suboptimal solution; then the initial solution space is constructed based on the suboptimal solution; finally, the optimal solution is found by operating the different behaviors of artificial bees in solution space. The proposed multiuser detector can make full use of the suboptimal solution and advantages of ABC to study the optimal value in the solution space. Simulation results have indicated that the BER performance, user capacity, and the NFE resistant ability of this novel algorithm are quite close to those of OMD, and they are also superior to those of MF, DEC, and MMSE. Furthermore, the convergence rate of SCM-ABC-MUD is better than that of ABC-MUD. And the computational complexity of the SCM-ABC-MUD is much lower than that of OMD.

## Figures and Tables

**Table 1:** Simulation parameters.

System	DS-UWB
Modulation mode	BPSK
Spreading codes (SC)	m sequences
The length of SC	255
Communication channel	AWGN
The number of testing information bits	3200000
The width of UWB pulse	0.8 ns
The pulse repetition period	*≈*2 ns
Limit	3
Initializing food source number	3

**Table 2:** The comparison of computational complexity using different MUD algorithms.

OMD	MMSE	DEC	SCM-ABC-MUD	MF
58.9	1.38	1.30	1.21	1
